# Distribution and transmission of copy number variations of uncertain significance in 105 trios

**DOI:** 10.1002/mgg3.2030

**Published:** 2022-08-09

**Authors:** Qiang Wen, Xiu Wang, Hao Zhang, Xiaoyan Liu, Zhihong Xu

**Affiliations:** ^1^ Department of Reproductive Genetics Deyang People's Hospital Deyang China; ^2^ Deyang Key Laboratory of Birth Defects Prevention and Control Deyang People's Hospital Deyang China

**Keywords:** copy number variation, CNVs of uncertain significance, distribution, heredity, prenatal counseling

## Abstract

**Background:**

The distribution and genetic characteristics of copy number variations (CNVs) remain unclear. Here, we investigated the distribution and transmission of CNVs of uncertain significance in fetuses.

**Methods:**

Low‐coverage massively parallels CNV sequencing of 105 families (parents and their fetuses) was performed to identify fetal CNVs of uncertain significance.

**Results:**

In the 105 fetuses, 176 CNVs of uncertain significance were detected, and the average number of CNVs carried by fetuses was 1.68 ± 0.80. Among the CNVs carried by the fetuses, 79.8% were inherited (~90.0% of the fetuses) and 20.2% were new mutations (~30.0% of the fetuses). We found that 58.9% CNVs were of maternal origin and 41.1% were of paternal origin. Among the CNV subtypes, de novo CNV distribution was significantly different from inherited CNV distribution. There was no difference in the distribution of maternal and paternal CNV subtypes in the fetuses. The proportion of microdeletions (36.7%) and microduplications (63.3%) was similar in the fetuses and parents. Furthermore, we found that when parents carried more CNVs of uncertain significance, the chance of passing them on to their offspring decreased.

**Conclusion:**

This study deepens our understanding of the genetic mechanisms associated with CNV transmission to assist clinicians in prenatal counseling.

## Graphical abstract

We found that most CNVs of uncertain significance were inherited from parents, and the proportion of maternal CNVs was higher. In the fetal CNVs of uncertain significance, there was a significant difference between the distribution of de novo CNVs and inherited CNVs subtypes. In addition, we hypothesized that the more CNVs carried by the parents, the lower the probability of transmission to the fetuses.

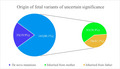

## 1 INTRODUCTION

The rapid development of genomics and molecular diagnostic technologies has led to the discovery and study of numerous human genetic diseases; however, information regarding novel genetic variations that have been revealed using advanced sequencing techniques remains limited (Klapwijk et al., 2021). Copy number variation (CNV) analysis using low‐coverage massively parallel CNV sequencing (CNV‐seq) has been used for prenatal diagnosis owing to the following advantages: a short and high‐throughput detection cycle, wide detection range, minimal nucleic acid requirement, and low cost. CNV‐seq can detect microdeletion or microduplication regions of more than 0.1 Mb, improving the sensitivity of CNV detection and increasing the detection rate of pathogenic CNVs (Liang et al., 2014). CNV‐seq, a whole‐genome random sequencing method with wide coverage, also detects variants of uncertain significance (VUS), which often complicates the content of prenatal genetic counseling (van der Steen et al., 2016).

In 2020, the American College of Medical Genetics and Genomics (ACMG) established specific classification criteria for CNVs and an evaluation system for interpreting their pathogenicity (Riggs et al., 2020). For instance, some CNVs in fetuses were downgraded from VUS to likely benign because trio analysis showed that the CNVs were inherited from healthy parents (Shi et al., 2019). In contrast, there have been cases of CNVs in trios (pregnant women, fetuses, and partner) being misclassified as VUS, likely pathogenic, or pathogenic using the ACMG CNV evaluation system, even after CNV‐seq and trio analysis, owing to the lack of phenotypic data. This results in increased financial cost, as well as anxiety and uneasiness among the parents, which are detrimental to fetal development (Lou et al., 2020).

Therefore, further investigation is needed to determine the benefits of performing trio analysis in the absence of phenotypic data, and further studies on the genetic mechanisms associated with CNVs are needed for their precise classification. The effective application of CNV‐seq in prenatal counseling requires extensive genotype–phenotype‐ and clinical case‐based databases to overcome the complexities of CNVs (i.e., gene penetrance, gene dose effect, gene interaction, and biochemical environment) and predict the clinical condition of the offspring (Fu et al., 2018). Therefore, this study was performed to explore the significance of CNV‐seq in trios and investigate the distribution and transmission of CNVs, thereby providing valuable data for improving the accuracy of prenatal counseling.

## 2 MATERIALS AND METHODS

### 2.1 Participants

The inclusion criteria for family subjects in this study were as follows: (1) pregnant women had undergone amniocentesis in the Prenatal Diagnosis Center of Deyang People's Hospital; (2) amniotic fluid was available for analysis using CNV‐seq, and the reported result indicated a fetus carrying VUSs; (3) both father and mother provided peripheral blood for CNV‐seq analysis in Deyang People's Hospital. The exclusion criteria were as follows: (1) the presence of maternal blood contamination in amniotic fluid samples; (2) the couple and the fetus were identified as unrelated lineages by short tandem repeat (STR) analysis; (3) the presence of only pathogenic or likely pathogenic CNVs as indicated by amniotic fluid analysis with CNV‐seq; (4) the presence of only benign or likely benign CNVs, by the same methodology. In total, 105 trios were included from September 2019 to March 2021. Among the 105 pregnant women, the indications for prenatal diagnosis included 23 high‐risk cases indicated by prenatal screening, 28 cases with structural abnormality indicated by ultrasound, 5 cases with an adverse pregnancy history, 18 cases of advanced maternal age, 4 cases with chromosomal abnormality indicated by non‐invasive DNA testing, 24 cases of voluntary testing due to medication during pregnancy and other reasons, and 3 cases with mixed indications. The age of the pregnant women and their partners ranged from 20 to 43 (average, 29.9 ± 5.0) years and 22 to 58 (average, 32.9 ± 6.8) years, respectively.

### 2.2 Biological samples

Amniocentesis was performed by a clinician to obtain 5 ml of fetal amniotic fluid from pregnant women. In addition, 5 ml of peripheral blood was collected from both parents through venipuncture. Genomic DNA was isolated using the DNeasy Blood & Tissue Kit (Qiagen Inc., Valencia, CA, USA) in accordance with the manufacturer's instructions. Genomic DNA quality and concentration were assessed using the Qubit 2.0 Fluorometer (Thermo Fisher Scientific, Waltham, MA, USA). Detection of maternal blood contamination in the amniotic fluid and identification of pedigree were performed using STR polymorphism linkage analysis with an ABI 3500Dx Genetic Analyzer (Applied Biosystems, Foster City, CA, USA). STR markers were used for chromosome 21 (21q11.2, D21S1411, D21S1412, D21S1414, D21S1433, and D21S1445), chromosome 18 (D18S1002, D18S391, D18S535, and D18S386), chromosome 13 (D13S305, D13S628, D13S634, and D13S742), and the sex chromosomes X and Y (DXS1187, DXS6809, DXS8377, DXS981, *AMELX*, *AMELY*, and *SRY*) (Microread Genetics Technology Co., Ltd., Beijing, China).

### 2.3 CNV‐seq and analysis

The DNA library was constructed according to a previously described method (Liang et al., 2014). Specifically, 50 ng of DNA was fragmented, and DNA libraries were constructed by end filling, adapter ligation, PCR amplification, and product purification. The DNA libraries were subjected to massively parallel sequencing on the NextSeq 500 platform (Illumina, San Diego, CA, USA) with an average sequencing depth of 0.10×. The quality control of the data was as follows: Q30 ≥ 85%, GC% within 38%–45%, Align rate ≥ 62.5%, and UR ratio ≥ 60%. AnnoroadPD software (Annoroad Gene Technology Co., Ltd., Beijing, China) was used to analyze the sequencing data, using the human reference genome GRCh37/hg19. Fetal CNVs with microdeletion or microduplication fragments longer than 100 kb were interpreted and classified. The classification was undertaken in accordance with the standards developed by the ACMG, in which CNVs are classified into the following five categories: pathogenic, likely pathogenic, uncertain significance, likely benign, and benign (Riggs et al., 2020). The CNVs were analyzed based on online public databases, including Online Mendelian Inheritance in Man (https://omim.org/), Database of Genomic Variants (http://dgv.tcag.ca/dgv/app/home), The University of California Santa Cruz Genome Browser (http://genome.ucsc.edu/), ClinGen (http://www.ncbi.nlm.nih.gov/projects/dbvar/clingen/), DECIPHER (https://www.deciphergenomics.org/), and relevant literature.

### 2.4 Statistical analysis

Only VUSs were included in the statistics. Continuous data are expressed as mean ± standard deviation, whereas count data are expressed as frequency (number) and percentage. The chi‐square test was used to compare the data from different demographic groups. All analyses were performed using SPSS 22.0 (IBM, Armonk, NY, USA). Statistical significance was set at *p* < .05.

## 3 RESULTS

### 3.1 Genetic source of VUSs

**FIGURE 1:**
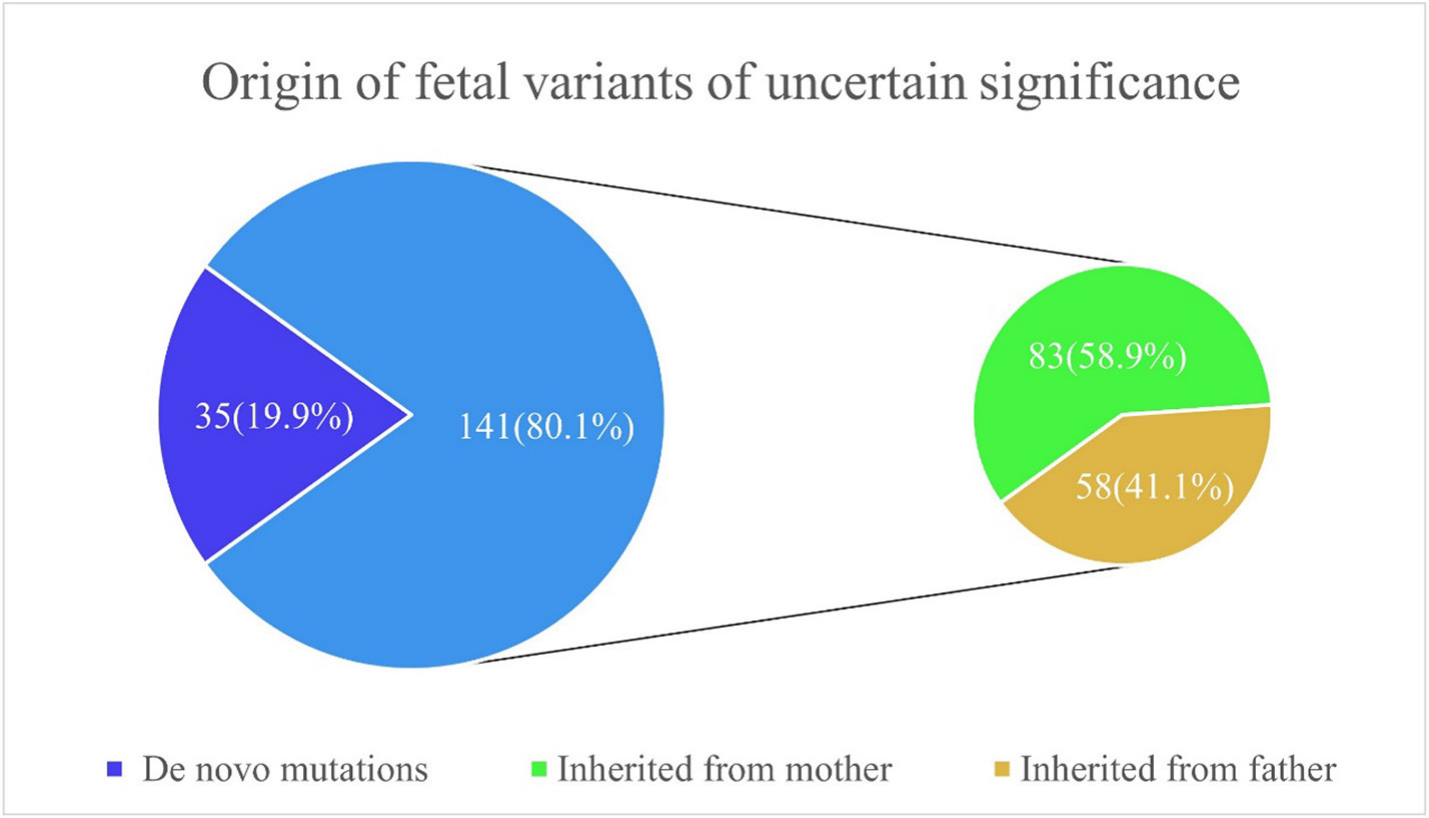
Origin of fetal variants of uncertain significance

In total, 176 VUSs were detected in the 105 fetuses, and the average number of VUSs carried by fetuses was 1.68 ± 0.80. Among them, 35/176 (19.9%) VUSs were de novo mutations and 141/176 (80.1%) VUSs were derived from parental transmission (Figure 1). Among the 105 fetuses (Supplementary Table S1), 32 (30.5%) carried de novo VUSs and 94 (89.5%) carried inherited VUSs. Approximately 90.0% of these fetuses carried parental‐origin VUSs; thus, the frequency of inherited VUSs was considerably higher than that of de novo VUSs (Table 1). De novo VUSs were the only type present in 11/105 (10.5%) fetuses, whereas 73/105 (69.5%) fetuses only harbored VUSs of parental origin. The remaining fetuses (21/105, 20.0%) had both de novo and parental VUSs. In addition, 83/141 (58.9%) VUSs were of maternal origin, whereas 58/141 (41.1%) VUSs were of paternal origin, with a ratio of approximately 3:2 (Figure 1). Therefore, the most common source of VUSs was inheritance, with a higher proportion of maternal sources than parental ones.

**TABLE 1:** Origin of variants of uncertain significance (VUSs) carried by 105 fetuses

Origin of VUSs	Fetuses (n)	Percentage (%)
Inherited from parents	94	89.5
De novo mutation	32	30.5
Only inherited from parents	73	69.5
Only de novo mutation	11	10.5

### 3.2 Distribution and transmission of VUS subtypes

In total, 18 microdeletions and 17 microduplications (approximately 1:1 ratio) were found among the de novo VUSs, whereas 45 microdeletions and 96 microduplications (approximately 1:2 ratio) were found among the inherited VUSs (Table 2). The chi‐square test showed that the distribution of fetal VUS subtypes was significantly different between new mutations and those inherited from parents (*p* = .039), whereas no significant difference was detected in the distribution of different subtypes of VUSs derived from each parent (*p* = .482). The proportions of microdeletions (35.8%) and microduplications (64.2%) in the fetuses were not significantly different from those in the parents (37.3% and 62.7%, respectively) or the trios (36.7% and 63.3%, respectively; Table 3). The transmission rate of the inherited microdeletions was non‐significantly lower than that of the microduplications (46.4% vs. 58.9%, *p* = .050; Table 4).

**TABLE 2:** Distribution of copy number variant types carried by fetuses

Origin	Microdeletions	Microduplications	*p*
De novo mutation	18 (51.4%)	17 (48.6%)	.039 < .05
Inherited from parents	45 (31.9%)	96 (68.1%)	
Inherited from mother	28 (33.7%)	55 (66.3%)	.482 > .05
Inherited from father	17 (29.3%)	41 (70.7%)	

*Note*: Data are n (%).

**TABLE 3:** Carrying of copy number variant types in the groups

VUSs	Microdeletions	Microduplications	*p*
Fetuses carrying	63 (35.8%)	113 (64.2%)	.757 > .05
Parental carrying	97 (37.3%)	163 (62.7%)	
Overall carrying condition	160 (36.7%)	276 (63.3%)	

*Notes*: Data are n (%). VUS, variants of uncertain significance.

**TABLE 4:** Delivery of copy number variant (CNV) types carried by parents

CNV types	Inherited to fetuses	Not inherited to fetuses	*p*
Microdeletions	45 (46.4%)	52 (53.6%)	.050
Microduplications	96 (58.9%)	67 (41.1%)	

*Note*: Data are n (%).

### 3.3 Transmission of VUSs harbored by parents

In this study, 260 VUSs were detected in parents, out of which 141 VUSs were transmitted to offspring, with a transmission rate of 54.2%, nearly 50%. The data regarding the probability of the transmission of VUSs from the parents to the fetuses are presented in Table 5. For parents carrying one VUS, the probability of VUS transmission to the fetus was 67.8%, which is higher than the hypothesized probability of 50.0% (based on the principle of independent assortment). For parents carrying two VUSs, the complete and partial transmission probabilities of the VUSs were 27.7% and 55.3%, respectively, which are higher than the hypothesized probabilities of 25.0% and 50.0%, respectively. However, for parents carrying three VUSs, the complete and partial transmission probabilities of the VUSs were 9.5% and 61.9%, respectively, which are lower than the hypothesized probabilities of 16.7% and 66.7%, respectively. Therefore, we suspected that the transmission probability tended to decrease with the increase in the number of VUSs carried by the parents.

**TABLE 5:** Delivery of variants of uncertain significance (VUSs) carried by parents to their fetuses

Number of VUSs carried^†^	All delivery	Partial delivery	No delivery	Total
1	59 (67.8%)	–^‡^	28 (32.2%)	87
2	13 (27.7%)	26 (55.3%)	8 (17.0%)	47
3	2 (9.5%)	13 (61.9%)	6 (28.6%)	21

*Note*: Data are n (%).

^†^ Parents carrying four VUSs were rare and were not included in the statistics.

^‡^ When carrying one VUS, there is no partial inheritance.

## 4 DISCUSSION

Although CNV‐seq is currently being used for prenatal diagnosis, the interpretation of results obtained using this technique is complicated, which limits its clinical application (Lou et al., 2020). In this study, we analyzed CNV‐seq data obtained from 105 trios; to the best of our knowledge, this is the first report regarding the distribution and transmission of VUSs in trios. Our data will help deepen our understanding of the genetic mechanisms associated with VUSs and improve the effectiveness of prenatal counseling.

Through the trio analysis of 105 pedigrees, we found that approximately 90.0% of these fetuses carried inherited VUSs and 80.0% of the carried VUSs were inherited from their parents. Briefly, the vast majority of fetal VUSs were of parental origin. In a previous study (Shi et al., 2019), the proportion of inherited CNVs in fetuses (72.3%) was markedly higher than that of de novo CNVs (27.7%). As the pathogenicity of CNVs was not classified in their statistics, the proportion of inherited CNVs was lower than that in our study, but still considerably higher than that of de novo CNVs. This is consistent with our results, showing that the trio analysis of families with VUSs cannot only trace the origin of the VUSs but also help clinicians to provide more accurate and effective clinical explanations and genetic counseling based on parental phenotypic information, which could greatly relieve parental fear and anxiety about VUSs. In addition, we found that 58.9% and 41.1% of the inherited VUSs were from female and male parents, respectively. Similarly, a previous study (Shi et al., 2019) reported that 59.8% of fetal CNVs were of maternal origin and 40.2% were of paternal origin. The parental analysis of inherited CNVs showed that fetuses acquired more maternal CNVs than paternal CNVs, suggesting that it is necessary for clinicians to collect detailed information about maternal phenotypes during prenatal filing, which could help obstetricians better report interpretation and provide genetic counseling during subsequent antenatal care.

In the comparison of the subtypes of VUSs, we found that the distribution of inherited VUS subtypes was significantly different from that of de novo VUSs. The proportion of microdeletions in the inherited VUSs was significantly lower than that in the de novo VUSs (Table 2). This means that microdeletions are more likely to be de novo than microduplications; in contrast, microduplications are more likely to be inherited, as shown in Table 4. The transmission rate of microdeletions carried by parents was lower than that of microduplications (46.4% vs. 58.9%). In a report on CNV analysis of fetal tissue following pregnancy loss (Chen et al., 2017), microdeletions were more frequent than microduplications. Because these CNVs were detected from aborted tissues, this result indirectly confirms that microdeletions are more likely to cause severe fetal developmental problems. Therefore, fewer microdeletions were detected among VUSs, and their proportion was lower than that of microduplications. Further parental analysis showed that there was a high proportion of microduplications in the VUSs inherited from parent to offspring, and there was no difference in the distribution of maternal and paternal VUSs in the fetuses (Table 2). This suggests that the inherited VUSs carried by fetuses, such as microduplications, had similar odds of paternal or maternal origin. Furthermore, it shows that the interpretation of clinicians' reports is challenging when conducting prenatal counseling for the parents of fetuses with VUSs, and the trio analysis could help clinicians make the right judgments (Klapwijk et al., 2021).

In addition, in this study, we found that the distribution of VUS subtypes carried by offspring was similar to that of VUS subtypes carried by parents; the proportion of microdeletions and microduplications was 36.7% and 63.3%, respectively (Table 3). As the parents were all of the childbearing age without an obvious clinical phenotype, this study considered that the sample was also representative of the population, to a certain extent. Therefore, it could be speculated that the distribution of VUS subtypes carried by healthy people of childbearing age was similar. However, owing to the sample size and sample inclusion criteria of this study, this result is bound to be revised or changed in future studies at a larger scale and with stricter sample inclusion criteria.

We hypothesized that parental VUSs would be randomly passed to their offspring; thus, the probability of each CNV being inherited was about 50%, and the average heritability of parental VUSs obtained in this study was 54.2%, nearly 50.0%. Upon testing this hypothesis, we found that the more the VUSs carried by the parents, the less likely that the VUSs would be passed on to the offspring (Table 5). This suggests that there was some protective selectivity in the parental distribution of their genetic material to their offspring. When healthy parents carry only one or two VUSs, the genetic mechanism treated the VUSs as less threatening, and the parents were more likely to pass them on to the offspring. However, when carrying multiple VUSs, owing to the complexity of the interaction between genes, the mechanism was more inclined to reject the transmission of VUSs to the offspring; evolutionarily, the benefit would be the avoidance of survival threats to the offspring. Of course, more research and clinical data are needed to explain and verify this phenomenon, and considerable work remains to be done on the genetic mechanisms around CNVs.

To the best of our knowledge, this is the first study to analyze the distribution and transmission of VUSs in trios, reveal the sources of fetal VUSs, and discuss the origin and proportions of microdeletions and microduplications. Nevertheless, this study had some limitations: (1) our cohort was established based on VUSs identified by CNV‐seq, making our conclusions applicable only to carriers of such VUSs; (2) the findings from our cohort were limited because of the limited budget available in our study; thus, a more extensive study is required to validate our results; (3) the VUSs may be identified as pathogenic or benign as genotype–phenotype databases expand and clinical data accumulates, affecting the current conclusions. Overall, further studies using large cohorts are needed to understand the genetic basis of CNV distribution and transmission, enabling to draw accurate, broad‐spectrum conclusions. CNV‐seq and other emerging technologies can help improve the effectiveness of prenatal counseling and prevent genetic diseases (Wang et al., 2018).

## 5 CONCLUSIONS

Overall, most fetuses carrying VUSs received them via parental inheritance, and maternal VUSs were more frequent than paternal VUSs. Among the subtypes of the VUSs, the distribution of inherited VUS subtypes was significantly different from that of de novo VUSs. However, there was no difference in the distribution of maternal and paternal VUSs subtypes in fetuses. Among the trios, the proportion of microdeletions and microduplications was 36.7% and 63.3%, respectively. In addition, we speculated that the more the VUSs carried by the parents, the more unlikely their transmission to the offspring. In conclusion, we analyzed the inheritance and distribution of VUSs in fetuses and trios. We provide clinical data and theoretical support for the exploration of genetic patterns and the characteristics of CNVs in the future and contribute to the in‐depth application of CNV‐seq technology in prenatal diagnosis.

## AUTHOR CONTRIBUTIONS

*Study design*: Wen Qiang. *Data collection*: Wen Qiang, Wang Xiu, and Liu Xiaoyan. *Data analysis*: Wen Qiang and Zhang Hao. *Manuscript preparation*: Wen Qiang and Xu Zhihong. All authors reviewed the manuscript.

## FUNDING INFORMATION

YYZX2021023: Chengdu University of Traditional Chinese Medicine.

## CONFLICT OF INTEREST

The authors declare no conflict of interest.

## ETHICAL CONSIDERATIONS

All participants provided written informed consent. The study was approved by the Prenatal Diagnostic Ethics Committee of Deyang People's Hospital.

## Supporting information


**Table S1** Distribution and origin details of VUSs in 105 trios

## Data Availability

The data that supports the findings of this study are available in the supplementary material of this article.
